# Medical and cardio-vascular emergency department visits during the COVID-19 pandemic in 2020: is there a collateral damage? A retrospective routine data analysis

**DOI:** 10.1007/s00392-022-02074-3

**Published:** 2022-08-05

**Authors:** Anna Slagman, Mareen Pigorsch, Felix Greiner, Wilhelm Behringer, Michael Bernhard, Jonas Bienzeisler, Sabine Blaschke, Volker Burst, Katharina Dechant, Michael Dommasch, Sebastian Ewen, André Gries, Felix Patricius Hans, Karl-Georg Kanz, Matthias Klein, Philipp Kümpers, Matthias Napp, Christopher Plata, Alexandra Ramshorn-Zimmer, Joachim Risse, Rainer Röhrig, Rajan Somasundaram, Domagoj Schunk, Felix Walcher, Thomas Walter, Dirk Weismann, Sebastian Wolfrum, Markus Wörnle, Yves Noel Wu, Martin Möckel

**Affiliations:** 1 Health Services Research in Emergency and Acute Medicine, Emergency and Acute Medicine CVK, CCM, Charité - Universitätsmedizin Berlin, Berlin, Germany; 2 Institute of Biometry and Clinical Epidemiology, Charité - Universitätsmedizin Berlin, Berlin, Germany; 3 Department of Trauma Surgery, Otto Von Guericke University Magdeburg, Magdeburg, Germany; 4 Department of Emergency Medicine, Medical University Vienna, Vienna, Austria; 5 Emergency Department, University Hospital of Düsseldorf, Heinrich-Heine-University, Düsseldorf, Germany; 6 Institute of Medical Informatics, University Hospital Aachen, Aachen, Germany; 7 University Medical Center Göttingen, Göttingen, Germany; 8 University Hospital Cologne, Cologne, Germany; 9 Friedrich-Alexander Universität (FAU), University Hospital Erlangen, Erlangen, Germany; 10 Technical University Munich, Munich, Germany; 11 University Hospital Homburg-Saar, Homburg, Germany; 12 Emergency Department, University Hospital Leipzig, Leipzig, Germany; 13 Medical Center, University Emergency Center, University of Freiburg, Freiburg, Germany; 14 Technical University Munich, Hospital Right of Isar, Munich, Germany; 15 Ludwig-Maximilians University Hospital Munich, Munich, Germany; 16 University Hospital Münster, Münster, Germany; 17 University Hospital Greifswald, Greifswald, Germany; 18 University Hospital Aachen, Aachen, Germany; 19 University Hospital Essen, Essen, Germany; 20 Emergency and Acute Medicine CBF, Charité - Universitätsmedizin Berlin, Berlin, Germany; 21 University Hospital Kiel, Kiel, Germany; 22 University Hospital Mannheim, Mannheim, Germany; 23 Department of Internal Medicine I, University Hospital of Würzburg, Würzburg, Germany; 24 Emergency Department, University Hospital Lübeck, Lübeck, Germany

**Keywords:** Collateral damage, Cardiovascular diagnoses, Emergency department, COVID-19, Pandemic

## Abstract

**Background:**

In this retrospective routine data analysis, we investigate the number of emergency department (ED) consultations during the COVID-19 pandemic of 2020 in Germany compared to the previous year with a special focus on numbers of myocardial infarction and acute heart failure.

**Methods:**

Aggregated case numbers for the two consecutive years 2019 and 2020 were obtained from 24 university hospitals and 9 non-university hospitals in Germany and assessed by age, gender, triage scores, disposition, care level and by ICD-10 codes including the tracer diagnoses myocardial infarction (I21) and heart failure (I50).

**Results:**

A total of 2,216,627 ED consultations were analyzed, of which 1,178,470 occurred in 2019 and 1,038,157 in 2020. The median deviation in case numbers between 2019 and 2020 was − 14% [CI (− 11)–(− 16)]. After a marked drop in all cases in the first COVID-19 wave in spring 2020, case numbers normalized during the summer. Thereafter starting in calendar week 39 case numbers constantly declined until the end of the year 2020. The decline in case numbers predominantly concerned younger [− 16%; CI (− 13)–(− 19)], less urgent [− 18%; CI (− 12)–(− 22)] and non-admitted cases [− 17%; CI (− 13)–(− 20)] in particular during the second wave. During the entire observation period admissions for chest pain [− 13%; CI (− 21)–2], myocardial infarction [− 2%; CI (− 9)–11] and heart failure [− 2%; CI (− 10)–6] were less affected and remained comparable to the previous year.

**Conclusions:**

ED visits were noticeably reduced during both SARS-CoV-2 pandemic waves in Germany but cardiovascular diagnoses were less affected and no refractory increase was noted. However, long-term effects cannot be ruled out and need to be analysed in future studies.

**Graphical abstract:**

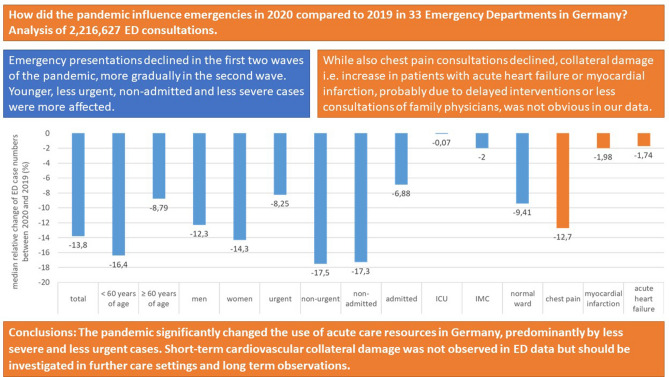

**Supplementary Information:**

The online version contains supplementary material available at 10.1007/s00392-022-02074-3.

## Introduction

During the first wave of the COVID-19 pandemic in 2020, emergency departments (EDs) in Germany and worldwide reported decreasing case numbers [1]. These reports were in line with previous observations from the 2003 SARS pandemic in Taiwan where decreased case numbers were especially observed in less urgent cases as well as traumatological presentations and pediatric cases [2]. These findings have been confirmed in other international studies, but primarily have been examined during the onset of the pandemic phase in 2020 [1, 3–7]. The main decrease in case numbers was again observed predominantly in non-urgent, non-admitted and younger cases [1].

However, also more severe diagnoses seemed to be decreasing along with the first pandemic wave: the phenomenon of decreasing case numbers was also observed for serious cardiovascular events such as myocardial infarction (MI) and stroke [1, 8, 9]. In a survey of the European Society of Cardiology, 70–90 percent of cardiologists worldwide reported having subjectively observed a delay in presentation to the ED or hospital in ST-elevation MI (STEMI) accompanied by a general reduction in cardiovascular presentations [9]. Further investigations confirmed these observations in different countries and additionally illustrated that case reduction was more pronounced in non-STEMI (NSTEMI) as compared to STEMI presentations [1, 10–12]. Based on the hypothesis of a consistent incidence of STEMI and NSTEMI, the term “cardiac collateral damage of the pandemic” was used and the main concern of cardiologists worldwide was the failure to provide life-saving care for heart attacks due to non-presentation at the hospital.

Even though the above-illustrated evidence might lead to a delayed or avoided presentation to medical services, the assumption of a steady incidence of MIs in the population could be a matter of debate. Even though the evidence is still lacking it could be speculated that change in lifestyle due to pandemic control measures like social distancing, hygiene measures, different working conditions and stress reduction might have led to decreased cardiovascular events in general and thus findings might be influenced also by aforementioned factors. On the other hand, contradictory results have been shown on increasing numbers of out-of-hospital cardiac arrests in specific regions which would further strengthen the hypothesis of “collateral damage” [13–17]. The first place of hospital-based clinical contact in case of suspected cardiovascular events is in most cases the ED. Thus, ED data might shed some light on the amount and relation of chest pain, suspected and confirmed MI presentations. Furthermore, avoided or postponed presentation during the first wave might have led to an increase of presentations with MI or long-term complications such as acute heart failure (AHF) in a later phase of the pandemic.

## Objectives

In this retrospective routine data analysis study, we aim to investigate the effects of the COVID-19 pandemic on ED visits in the whole year of 2020 as compared to the previous year in all ED presentations and in several subgroups with a special focus on the second pandemic wave in general and specifically in cardiovascular chief complaints and diagnoses.

## Methods

### Study centers

A total of 33 German study sites (24 university hospitals and 9 non-university hospitals) participated in this nationwide retrospective routine data analysis. Anonymized aggregated data was collected from various electronic health record systems in the respective EDs.

### Setting

Data collection took part in January and February 2021. At 22 study sites, case numbers were extracted from the respective Emergency Department Information Systems (EDIS) directly and collected in excel sheets in an anonymous, aggregated from. Aggregated data were then transferred directly to Charité—Universitätsmedizin for analysis. In 11 hospitals, the data was retrieved via the AKTIN Emergency Department Data Registry, that enables data protection-compliant multi-center use of standardized routine data from EDs [18]. After a positive vote from the AKTIN data use and access committee (Project ID 2021-001), data query was carried out with subsequent processing and aggregation in the AKTIN trusted data analyzing center and then transferred to Charité—Universitätsmedizin Berlin.

### Participants

All visits of patients who attended the participating EDs within the observation period 2019 or 2020 were analyzed. As anonymization was given by the nature of the data quest no informed consent was required.

### Variables

We collected aggregated data per calendar week of every ED visit of the participating EDs within the years 2019 and 2020 including the main diagnosis (ICD-10), age, gender, urgency (Manchester triage system or emergency severity index: less urgent = triage scores 4 and 5, urgent = triage scores 1, 2 and 3), the modality of discharge from the ED (home or admission to hospital) and the designated hospital ward (intensive care unit-ICU, intermediate care unit-IMC, normal ward). Myocardial infarction was assessed as ICD-10 codes I21.0-I21.4. Further extracted diagnoses were heart failure (I50; operationalized as acute heart failure in this manuscript), unstable angina (I20), chest pain (R07), and any diagnosis of I21 when specified as ‘excluded’ in the ED.

### Data synthesis and analysis

The method of data extraction and transfer has been previously described in more detail [1]. In brief, anonymous, aggregated data were sent to the central data management at Charité—Universitätsmedizin Berlin. Data validity was checked at the study sites and centrally at Charité—Universitätsmedizin Berlin for each clinic before data synthesis and analysis. The excel files (.xlsx) of the individual clinics were then transferred to SPSS (IBM, Version 26) and merged into one data set.

The data were analysed using the statistics program R version 4.5 [19]. Median case numbers and interquartile ranges (IQR) over all EDs for 2019 and 2020 as well as relative deviations for 2020 in relation to 2019 were calculated. Median deviation per clinic between year 2019 and 2020 with a 95% confidence interval (95% CI) was calculated for presentations and different diagnoses using the function groupwiseMedian() with the percentile method, which is a bootstrap method, from the R package rcompanion [20]. Variables with low frequencies were aggregated over 4 weeks, starting with calendar week 1–4. Boxplots were created to visualize absolute values for 2019 and 2020 and relative deviations between 2020 and 2019. Due to different numbers of days in the first calendar week between 2019 and 2020, weekly presentations start in week 2. In addition, the maximum median change with 95% CI was calculated. The pandemic phases were defined according to Schilling et al. and the first pandemic wave was defined from CW 10 to 20 and the second pandemic wave from CW 40 until the end of 2020 [21].

### Ethics, data protection and security

In this study, pooled, aggregated and anonymous data were used. The data protection officers of the Charité—Universitätsmedizin Berlin were consulted in an advisory capacity. The project was approved by the institutional review board of the Charité—Universitätsmedizin Berlin (EA1/163/20) and the data use and access committee of AKTIN (Project-ID 2021-001).

## Results

**Fig. 1:**
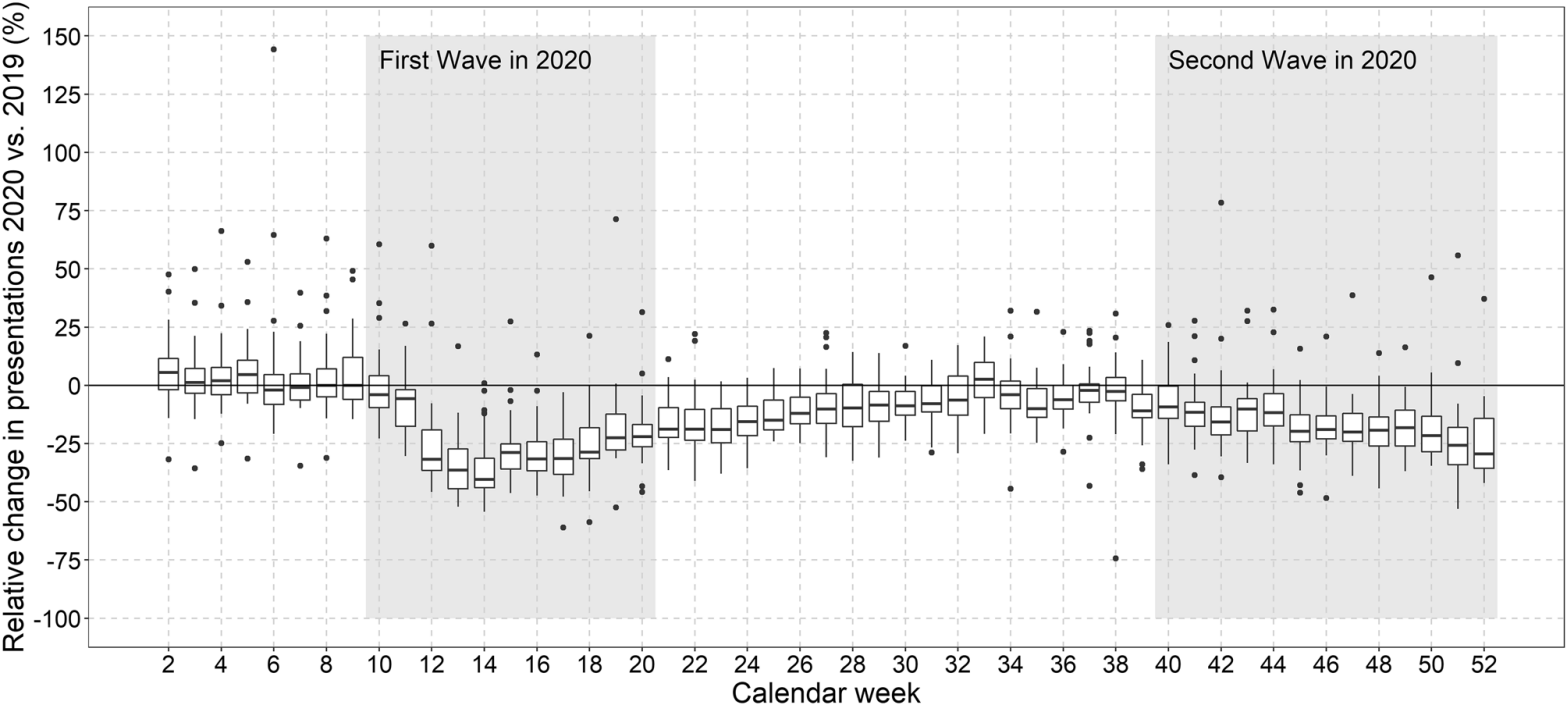
Relative change (%) in all, non-selected, ED presentations 2020 as compared to 2019 per calendar week. *N* = 5 outliers above 150% relative change are not depicted in Fig. 1

In total, 2,216,627 presentations from 33 EDs were analyzed, whereof 1,038,157 took place in 2020 and 1,178,470 in 2019. The median case number over all EDs was 694 (IQR: 385-841) in 2019 and 604 (IQR: 348-743) in 2020 (Supplement Table 1). The median deviation in case numbers per clinic between 2019 and 2020 was − 14% (CI – 11–− 16%). After a marked drop in all ED visits in the first COVID-19 wave in spring 2020, case numbers normalized in summer and started to decline again in calendar week 39, 2020. Until the end of the year 2020 case numbers constantly decreased (Fig. 1).

The relative reduction of ED presentations was more pronounced in women and in younger patients. Relative reductions occurred to a lesser extent in older patients (maximum median change in older patients − 34%, 95% CI − 37%–− 32% in CW 14; in younger patients − 42%, 95% CI − 47%–− 40% in CW 14). An earlier re-incline of case numbers was observed in older patients, already starting from calendar week 16, and relative reduction was less pronounced during the second COVID-19 wave in autumn 2020 (maximum median change in older patients of − 18%, 95% CI − 24%–− 9% in CW 52; in younger patients of − 35%, 95% CI − 41%–− 30% in CW 52) (Supplement Figure 1 and 2).

### Clinical features

All categories of urgency as assessed by triage scores were affected, but less urgent case numbers decreased more pronounced and increased delayed as compared to more urgent ED presentations (Supplement Figure 3). The decrease in urgent ED presentations was also less affected during the second wave. The same was true for the comparison of non-admitted and admitted ED presentations (Supplement Figure 4): while non-admitted (outpatient) ED presentations declined to a greater extent (maximum median change in non-admitted of − 46%, 95% CI − 50%– − 43% in CW 14) than admitted ED presentations during the first pandemic wave, still also admitted cases decreased by a maximum median change of − 29% (95% CI − 34%– − 18%) in CW 14. Again, the decline was less pronounced predominantly in admitted patients during the second pandemic wave.

Differences were also observed with respect to the level of care for patients with in-hospital treatment (admitted patients; Supplement Figure 5): while admissions to the normal ward showed a sharper decline during the first pandemic wave and stayed low or comparable to the previous year over the whole year of 2020, admissions to intermediate care (IMC) and intensive care units (ICU) from the EDs declined to a lesser extend during the first pandemic wave (maximum median change in the normal ward of − 22%, 95% CI − 29%– − 17%; in IMC of − 9%, 95% CI − 48%–0%; in ICU of − 11%, 95% CI − 19%–0% in CWS 13–16) and were even higher as compared to the previous year during the summer months (maximum median change in IMC of 14%, 95% CI − 18–41 in CWs 29–32; in ICU of 11%, CI − 1%–46% in CWs 25–28). Little relative reduction was observed during the second wave (maximum median relative change in the normal ward of − 14%, 95%CI − 23%–− 10% in CWs 49–52, in IMC of − 7%, CI − 39%–16% in CWs 49–52; in ICU of − 6%, CI − 17%–0% in CWs 45–48).

## Diagnoses

In the entire observation period admissions for chest pain, myocardial infarction and heart failure were less affected but also decreased as compared to the previous year. No clear trend toward increasing case numbers after the reduction of case numbers in the first pandemic wave was observed.

### Chest pain

**Fig. 2:**
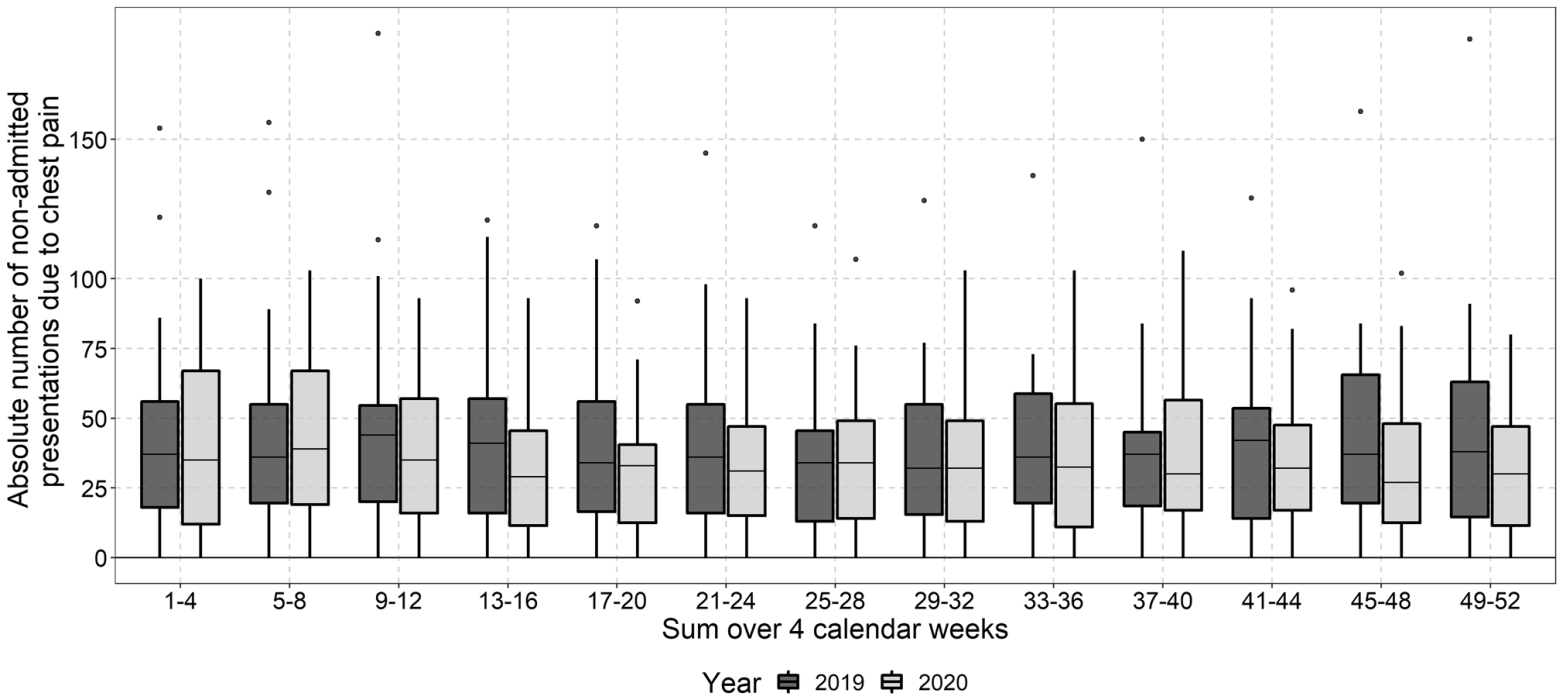
Absolute number of ED presentations with chest pain (I20, R07, I21-exclusion) aggregated over 4 weeks in 2020 and 2019. Data of 27 EDs were available for these analyses (median 36; IQR: 17–59; *n* = 13,587 presentations in 2019 and median 32; IQR: 14–52; *n* = 11,434 in 2020)

The median relative change of ED presentations suggestive of MI (chest pain presentations) was − 13% (95%CI − 21%–2%) over the whole observation period while median absolute numbers were declined only slightly, mainly between CW 9 and 16 and during the second pandemic wave between CW 37 and 44 (Fig. 2). There was no increase of chest pain ED presentations after these time frames and numbers were comparable to the previous year.

### Myocardial infarction

**Fig. 3:**
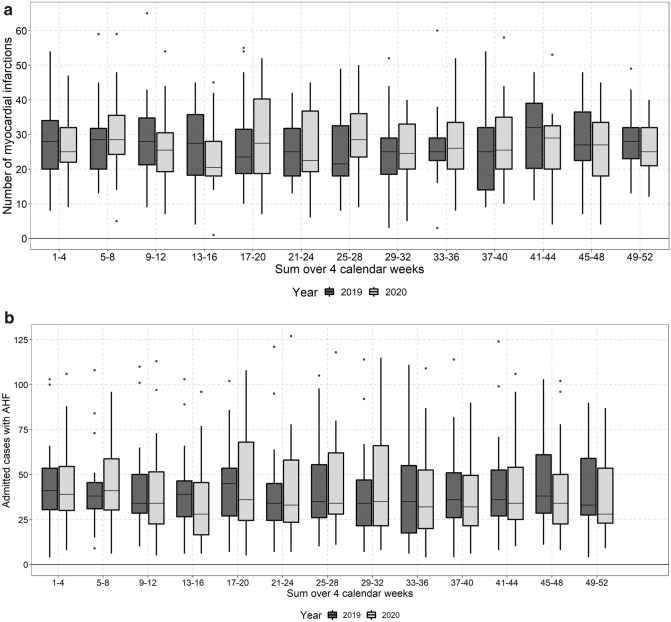
**a** Absolute number of ED presentations with myocardial infarction (I21.0-I21.4.) aggregated over 4 weeks in 2020 and 2019. Data of 20 EDs were available for these analyses. The median number of presentations was 26 (IQR: 19–34) in 2019 (*n* = 6500) and 26 (IQR: 20–34) in 2020 *n* = 6,788. **b** Absolute number of ED presentations who were admitted to the hospital with acute heart failure (I50) aggregated over four weeks in 2020 and 2019. Data of 20 EDs were available for these analyses. There median number of cases was 38 (IQR: 26–55) in 2019 (*n* = 13,480) and 35 (IQR: 23–64) in 2020 (*n* = 13,164)

The median relative change of MIs over the whole observation period was − 2% (95%CI − 9%–9%). Median absolute numbers of MI were also lower in 2020 as compared to 2019 in the CW 13–16 but seemed to incline in CW 17–20 (Fig. 3a).

### Acute heart failure

Regarding acute heart failure (AHF) the median relative change over the whole observation period was again − 2% (95% CI − 10%–6%). The median absolute number of AHF presentations to the ED declined substantially in CW 13–20 and a smaller decline was observed in the second pandemic wave in Europe again (Fig. 3b). During the overall pandemic period no increase in AHF presentation was observed in comparison to 2019 and median case numbers were only slightly higher in the beginning of the year 2020 (CW 5–8).

## Discussion

In this observational, retrospective multicenter study analyzing German ED presentations during the SARS-CoV-2 pandemic year 2020 in comparison to the previous year (2019), we were able to illustrate that ED presentations, in general, declined more pronounced during the first pandemic wave in spring 2020 as compared to the second pandemic wave in autumn and winter 2020. The reduction was more pronounced in younger, less-urgent, non-admitted cases and in admissions to normal wards during both pandemic waves. However, more severe cases (older age, urgent and admitted cases) as well as admissions to IMC or ICU increased earlier and were less or even not at all reduced during the second pandemic wave. A further interesting finding was the increase of ICU admissions during the summer months of 2020, which might hint at some sort of collateral damage like postponed elective procedures or aggravation of disease due to delayed treatment.

Our observations are in line with other international data showing a decrease in general ED presentations during the first pandemic wave but shed further light into the trend of ED admissions after the initial reduction and during the second pandemic wave and onwards in 2020 [1, 3–7, 22–25].

Regarding cardiovascular diseases, ED presentations for chest pain, unstable angina or MI-exclusion declined slightly. Admissions to hospital with MI decreased during the first pandemic wave but increased again to comparable numbers in 2019 in the following CWs. This could be a sign of postponed ED presentation or collateral damage. These findings are in line with other studies reporting decreased numbers of hospital admissions and ED presentations with acute coronary syndrome and MI during the first pandemic wave [10–12, 16, 26–29]. There was no clear trend for a compensatory increase after the initial lockdown in 2020. However, MI numbers in our study also showed some alterations in the following CWs, which illustrates that these results might be caused by smaller case numbers in general and thus should not be over interpreted. Also hospital admissions for AHF, which might have been an indicator for missed MIs during the first pandemic wave, remained constant and thus the current data do not support the hypothesis of massive collateral damage to cardiovascular patients due to COVID-19 [9–11, 13]. Nevertheless, it has to be taken into account that delayed ED presentations of patients with coronary artery disease might have long-term consequences over years rather than effects that can be seen within several months. In addition, evidence shows increased numbers of out-of-hospital cardiac arrests (OHCA), especially in high COVID-19 incidence regions, while areas with lower COVID-19 incidence were less affected. [13–16, 30]

Several causative factors have been discussed and it is still unclear, whether the care-seeking behavior of patients was affected by the fear of COVID-19 infection in the hospital, whether social isolation measures triggered the absence of care-giving persons who usually would initiate hospital admission has partly caused this phenomenon, or whether there might also be a direct association between COVID-19 infection and cardiac arrest [17]. Nab et al. investigated reasons for delayed care-seeking behavior in Dutch ED patients and beneath the fear of a COVID-19 infection, also the ‘misperceptions of the accessibility of (ED) services and the legitimacy for seeking emergency care’ in times of sparse health care resources were reported as main drivers for avoiding ED consultancies in the first place [31]. Therefore, further research is necessary to investigate causal factors for non- and delayed care-seeking behavior and monitor cardiovascular morbidity over the following years. Despite that, also out of hospital mortality for other diseases such as neoplasms and endocrine diseases has been increased during the pandemic and warrants further investigation [32]. In the shed of these data it could be recommended that health information, in general, should clearly emphasize seeking care whenever patients experience severe symptoms or aggravation of the disease, also in times of sparse health care resources and high incidence of COVID-19 or other pandemic diseases.

However, it also needs to be mentioned that there is still too little evidence to interpret whether the reduced number of cases, especially of serious diseases as observed by several studies can be explained exclusively by a delayed or absent consultation of utilization of medical care structures [1–5, 8–12]. This hypothesis includes the implicit assumption of a constant incidence of cases which needs to be carefully investigated since it remains unclear whether the reduced number of ED visits may also be explained by declining incidences of certain diseases. Favorable environmental conditions and general hygiene measures, for example, most likely have reduced the number of respiratory infections in general [33]. Also infection-driven and other exacerbations of chronic diseases which might have otherwise required urgent treatment may have been reduced.

The moderate increase of ICU admissions from the ED during the summer months of 2020 is uncommon and warrants further investigation. Different factors should be discussed in the context of ICU admissions from the ED: first, there could have been an effect regarding the presenting ED cases (in-flow) but also alterations in hospital and ICU capacities need to be addressed (out-flow). Regarding the “in-flow” of ED cases its important to mention that in general, ED case numbers in the summer period are not much lower than in winter, but the severity of cases is usually lower with, for example, an increase in milder accidents and decreased numbers of severe respiratory infections. This case mix might have been affected by the first pandemic wave which might hint at some sort of collateral damage leading to more severe hospital cases than usually. Little is yet known about the impact of reducing inpatient services to clearing up capacities for COVID-19 cases on patients with other diagnoses than COVID-19 [34]. The elective procedures and prevented inpatient admissions which had to be postponed could also have led to increased use of other care providers, such as EDs, over the long-term and might result in an increase in ICU admissions from EDs.

Furthermore, there might be an effect of “staying at home” (in Germany) during the summer month of 2020, which might have led to alterations in the case mix. While especially some parts of the older population in Germany who tend to travel during the summer months might have reduced traveling in 2020 and could have presented to German EDs, which might have otherwise been treated elsewhere. Still there could also be an effect of non-presentation to EDs with milder symptoms, which might have resulted in disease progression over time and led to more severe cases during the summer months. The “out-flow” from EDs to ICUs is also affected by ICU capacities and should not be confused with overall ICU-admission rates.

Interestingly, data from German ICUs show a decrease in ICU admission and mechanical ventilation in COVID-19 patients in the second pandemic wave in comparison to the first pandemic wave [35]. Thus, the increase in ICU admissions is unlikely to be driven by COVID-19 infections and underlying causes need to be investigated in non-aggregated data allowing for further statistical procedures.

### Limitations

This study investigates case numbers during the COVID-19 pandemic in a relatively large number of EDs in Germany. This sample could, however, still not be regarded as representative for all German EDs since mainly large, tertiary care clinics and predominantly university hospitals were investigated. Even though there is no hypothesis supporting the assumption of differing observations in smaller hospital EDs, there could still have been a trend to less severe presentations to smaller hospitals to avoid crowding of larger tertiary care hospitals, also to keep resources for urgent care of COVD-19 cases. This could be an effect regarding self-presentation but could also be true for medical transports and this limitation should be kept in mind when interpreting the results. Moreover, not all participating hospitals were able to provide data for all investigated subgroups over both years of observation. Thus, subgroup analyses are often based on a smaller number of hospitals. Regarding the analysis of certain ICD-codes as main hospital diagnosis it is worth mentioning that the investigated ICD-codes could have been coded as a secondary diagnosis while only main diagnoses were analysed in this study and thus the presented numbers could be biased in the direction of under-estimation. Still the comparison of cases with certain diagnoses between calendar years could be regarded as being valid since the above-mentioned potential bias can be assumed to have occurred in both years of the observation period.

## Conclusions

In Germany, the COVID-19 pandemic led to a marked drop in medical presentations to the nation’s EDs in the first and second wave of the pandemic, consistent in the whole country. The drop was more pronounced in less severe ED presentations, especially during the second wave. There were no clear signs of collateral damage regarding cardiovascular diseases even though long-term effects may occur only in the next years and need to be further monitored. In contrast, an unexpected increase in ICU admissions during the summer months of 2020, when COVID-19 admissions were low, could be a sign of collateral damage leading to severe hospital cases but the underlying causes and conditions require further investigation.

## Supplementary Information

Below is the link to the electronic supplementary material.

Supplementary file1 (DOCX 1097 KB)

## Data Availability

Anonymized research data are available from the corresponding author upon reasonable request.
