# Supplementary material for: Medical and cardio-vascular emergency department visits during the COVID-19 pandemic in 2020: is there a collateral damage? A retrospective routine data analysis
Source: Clin Res Cardiol. 2022 Aug 5;111(10):1174–82. doi: 10.1007/s00392-022-02074-3 (PMC9362706; doi:10.1007/s00392-022-02074-3)
Supplement: Supplementary file 1 — Supplementary file1 (DOCX 1097 KB) [file 392_2022_2074_MOESM1_ESM.docx]

Anna Slagman^1^, Mareen Pigorsch^2^, Felix Greiner^3^, Wilhelm Behringer^4^, Michael Bernhardt^5^, Jonas Bienzeisler^6^, Sabine Blaschke^7^, Volker Burst^8^, Katharina Dechant^9^, Michael Dommasch^10^, Sebastian Ewen^11^, André Gries^12^, Felix Patricius Hans^13^, Karl-Georg Kanz^14^, Matthias Klein^15^, Philipp Kümpers^16^, Matthias Napp^17^, Christopher Plata^18^, Alexandra Ramshorn-Zimmer^12^, Joachim Risse^19^, Rainer Röhrig^20^, Rajan Somasundaram^21^, Domagoj Schunk^22^, Felix Walcher^3^, Thomas Walter^23^, Dirk Weismann^24^, Sebastian Wolfrum^25^, Markus Wörnle^15^, Yves Noel Wu^1^, Martin Möckel^1^

***Supplement Table 1*** *Median case numbers per ED and median relative change over the years 2019 and 2020*

|  | **median case number per CW per ED** | | **median relative change over both years** | | |  |
| --- | --- | --- | --- | --- | --- | --- |
|  | **median (IQR) 2019** | **median (IQR) 2020** | **median value** | **lower 95%-CI** | **upper 95%-CI** | **number of EDs (n)*** |
| **total** | 694  (385-842) | 604  (348-743) | -13.8 | -15.4 | -8.5 | 33 |
| **<60 years of age** | 406  (221-546) | 337  (192-457) | -16.4 | -18.9 | -13.2 | 33 |
| **≥ 60 years of age** | 264  (155-326) | 238  (149-298) | -8.8 | -11.5 | -6.5 | 33 |
| **men** | 367  (188-444) | 318  (179-400) | -12.3 | -14.4 | -8.1 | 33 |
| **women** | 327  (192-403) | 283  (173-354) | -14.3 | -16.7 | -10.5 | 33 |
| **urgent** | 308  (178-433) | 276  (170-381) | -8.3 | -11.8 | -4.3 | 32 |
| **less-urgent** | 270  (145-403) | 218  (113-327) | -17.5 | -21.5 | -11.7 | 32 |
| **non-admitted** | 369  (176-496) | 306  (175-418) | -17.3 | -19.9 | -13.2 | 33 |
| **admitted** | 208  (120-285) | 195  (125-253) | -6.9 | -8.2 | -2.9 | 33 |
| **ICU** | 19  (9-45) | 22  (10-45) | -0.1 | -2.8 | 7.9 | 32 |
| **IMC** | 19  (5-35) | 15  (4-33) | -2.0 | -13.0 | 16.9 | 15 |
| **normal ward** | 162  (110-208) | 143  (94-191) | -9.4 | -14.5 | 3.4 | 15 |
|  | **median case number per 4 CWs per ED** | | **median relative change over both years** | | |  |
|  | **median (IQR) 2019** | **median (IQR) 2020** | **median value** | **lower 95%-CI** | **upper 95%-CI** | **number of EDs (n)** |
| **chest pain** | 36 (17-56) | 32 (14-52) | -12.7 | -20.8 | 2.1 | 27 |
| **MI** | 26 (19-34) | 26 (20-34) | -2.0 | -8.6 | 10.7 | 20 |
| **AHF** | 38 (26-55) | 35 (23-64) | -1.7 | -9.6 | 6.0 | 20 |

***Abbreviations:*** *AHF – acute heart failure, CI – confidence interval, CW – calendar week, ED – emergency department, IQR – inter quartile range, MI – myocardial infarction, *for calculation of median relative changes over both years*

***Supplement Figure 1*** *Relative change of ED presentations in 2020 as compared to 2019 stratified by gender*

*
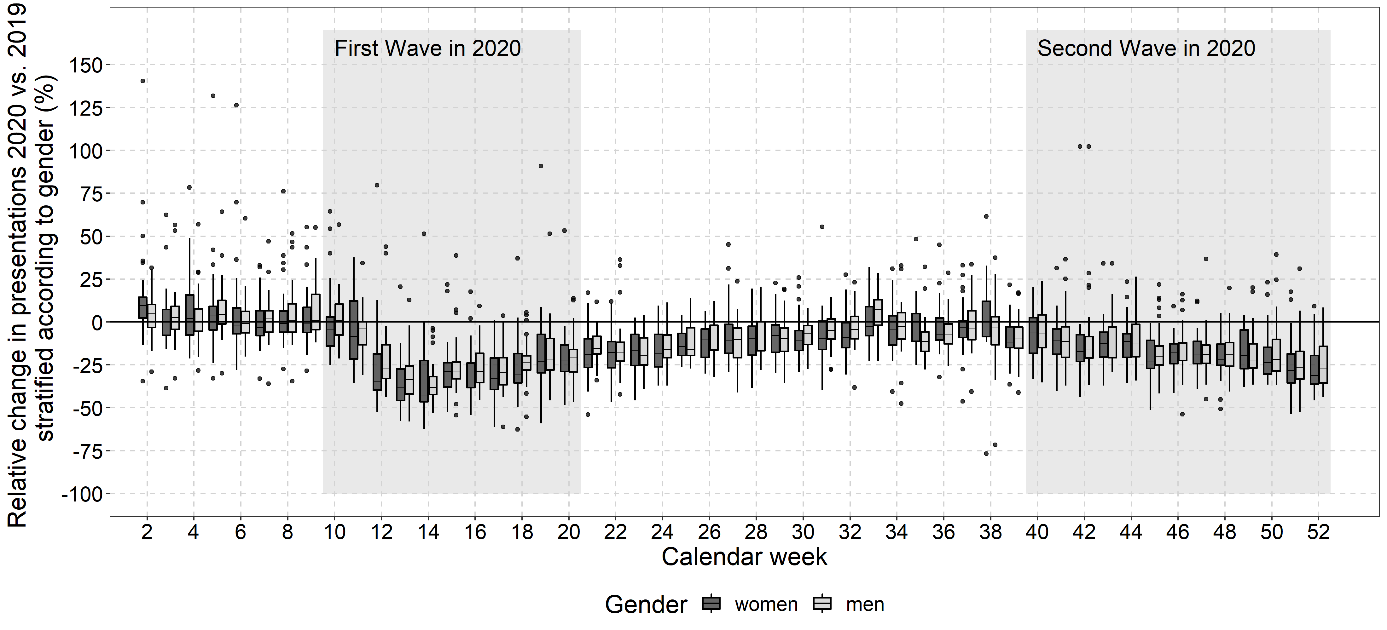
*

*Legend: The median number of cases over all EDs was 327 (IQR: 192-403) for women in 2019 (n=567,010) and 283 (IQR: 173-354) in 2020 (n=495,010), while there were in median 367 (IQR: 188-444) cases of men in 2019 (n=617,151) and 318 (IQR: 179-400) in 2020 (n=546,115). n=9 outlier above 150% relative change are not shown.*

***Supplement Figure 2*** *Relative change of ED presentations in 2020 as compared to 2019 by age categories (<60 years of age vs. ≥ 60 years of age)*

*
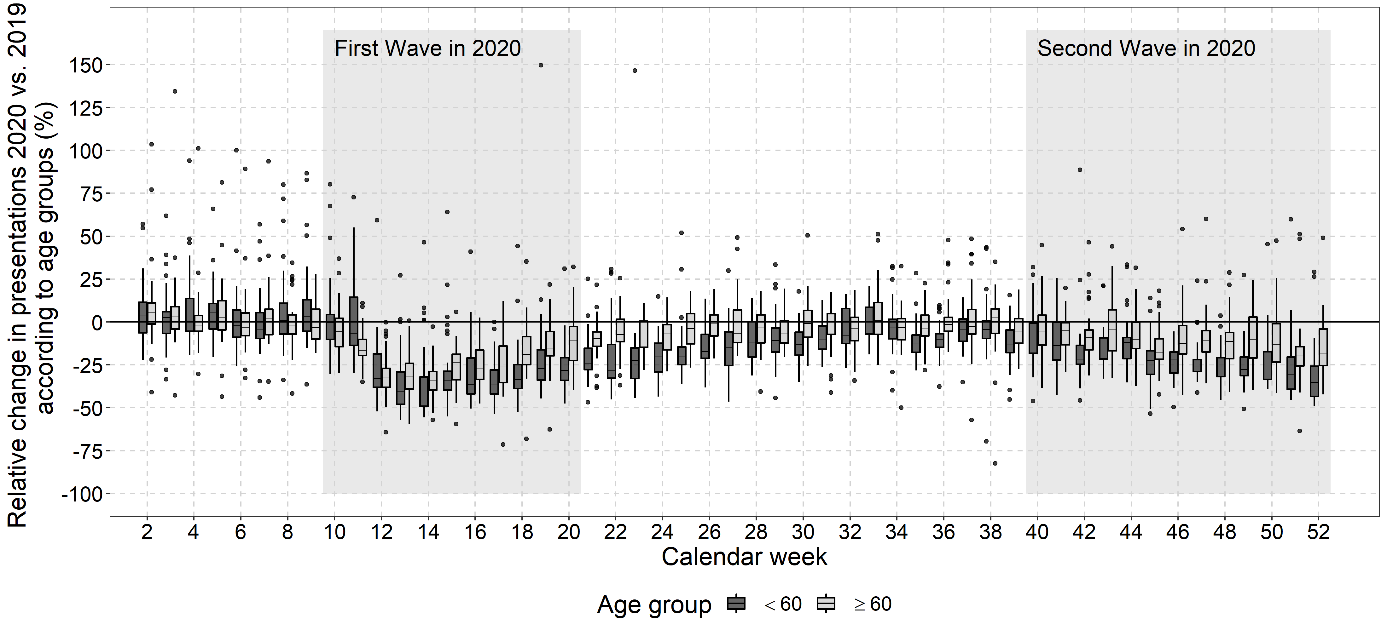
*

*Legend: The median number of presentations of patients below 60 years of age over all EDs was 406 (IQR: 221-546) in 2019 (n=723,481) and 342 (IQR: 195-460) in 2020 (n=611,484). Regarding patients at or above 60 years of age the median number of presentations was 264 (IQR: 155-326) in 2019 (n=455,907) and 238 (IQR: 149-298) in 2020 (n=417,694) were analysed. n=7 outlier above 150% relative change are not shown.*

***Supplement Figure 3*** *Relative change of ED presentations in 2020 as compared to 2019 in urgent as compared to less urgent cases according to the initial triage category in the ED*

*
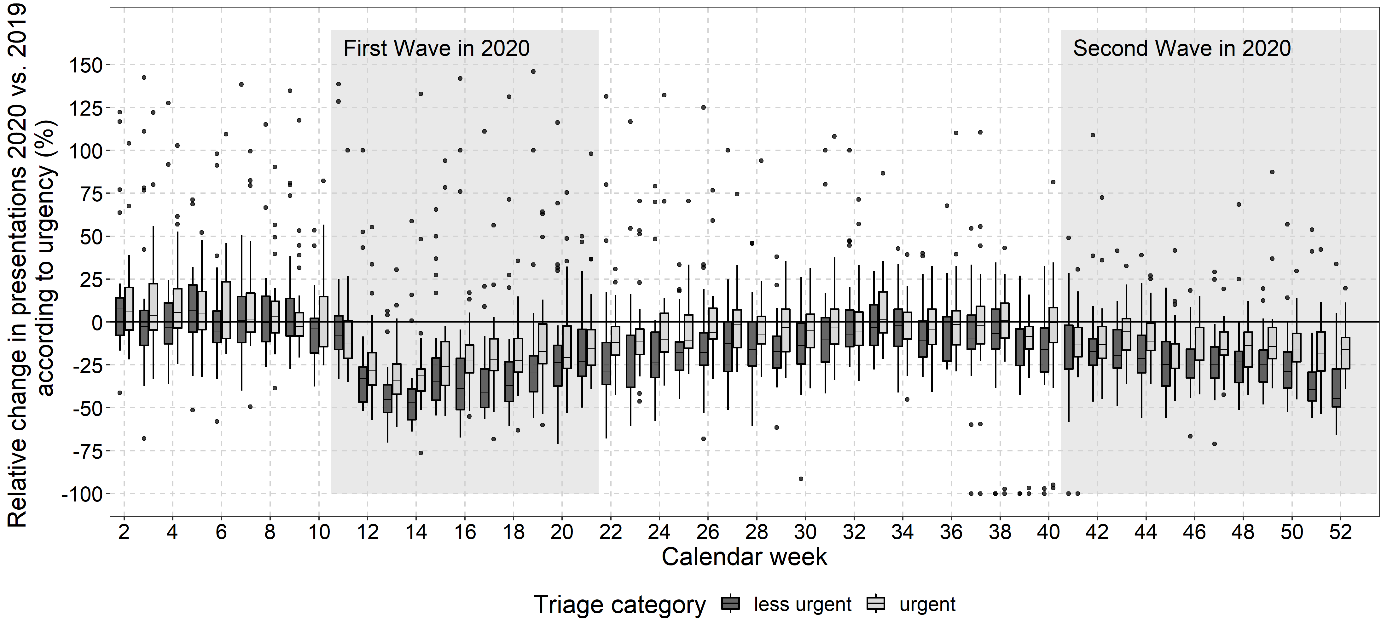
*

*Legend: The median number of presentations with urgent triage categories was 308 (IQR: 178-433) in 2019 (n=539,602) and 276 (IQR: 170-381) in 2020 (n=503,041) while in non-urgent triage categories the median case number was 270 (145-403) in 2019 (n=466,013) and 218 (IQR: 113-327) in 2020 (n=387,190). n=72 outlier above 150% relative change are not shown.*

***Supplement Figure 4*** *Relative change of ED presentations in 2020 as compared to 2019 in patients whose ED presentation led to admission to hospital (admitted) and in patients who were discharged directly from the ED (non-admitted)*

*
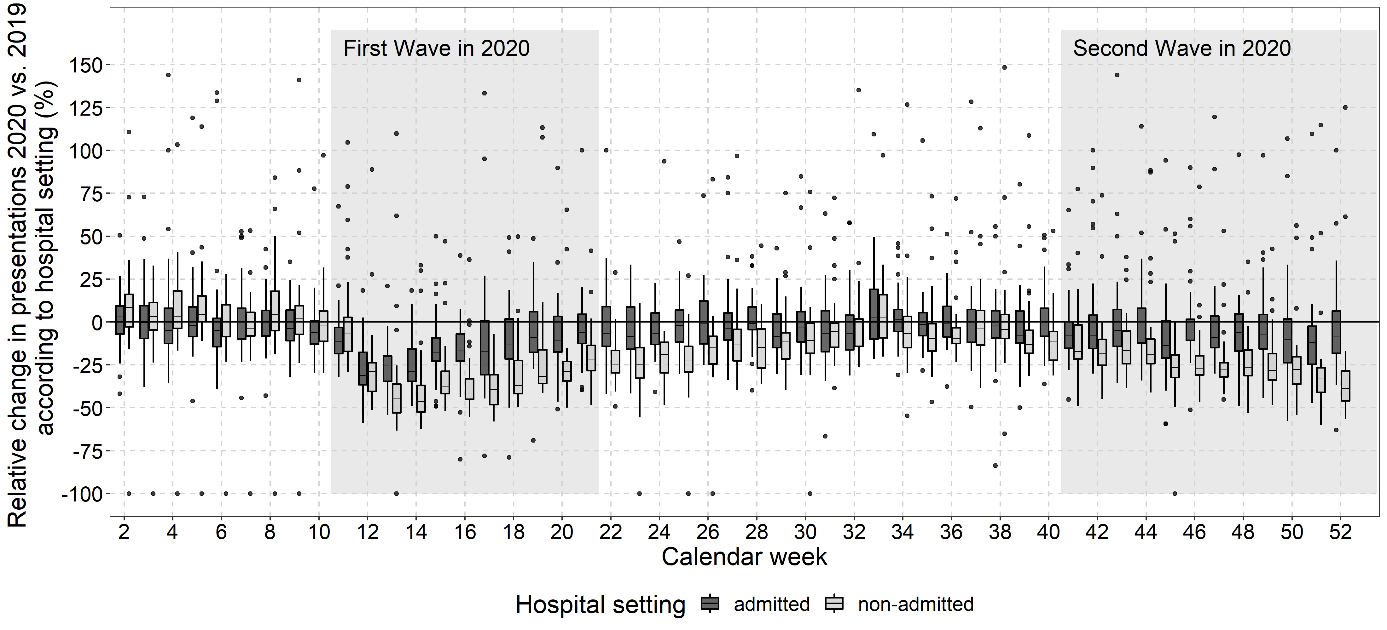
*

*Legend: A median number of 369 (IQR: 175-496) non-admitted cases was analysed in 2019 (n=627,264) and 306 (IQR: 175-418) in 2020 (n=530,981), while in median 208 (IQR: 120-285) admitted cases were analysed in 2019 (n=377,602) and 195 (IQR: 125-253) in 2020 (n=355,220). n=35 outlier above 150% relative change are not shown.*

***Supplement Figure 5*** *Relative change of ED presentations in 2020 as compared to 2019 in admitted patients in categories of different admission wards*

*
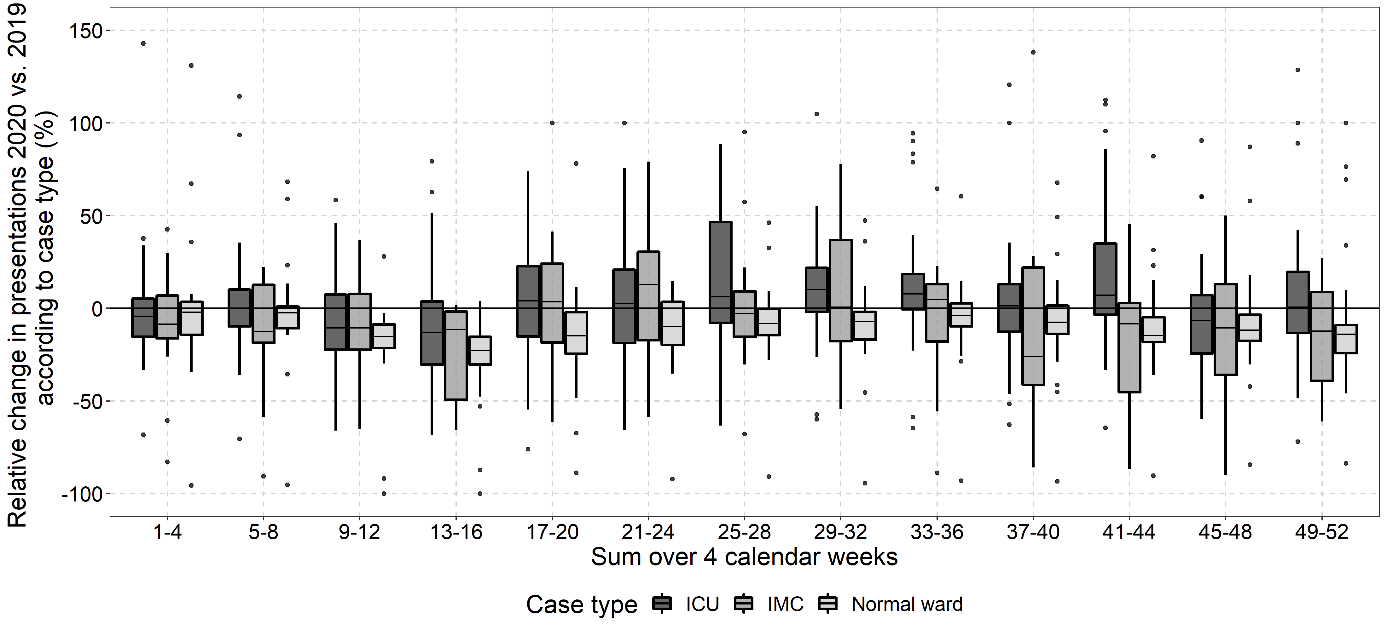
*

*Legend: Normal ward (median 162; IQR: 110-208; n=284,348 in 2019 and median 143; IQR: 94-191; n=251,094 in 2020), intermediate care (IMC; median 19; IQR: 5-35; n=25,719 in 2019 and median 15; IQR:4-33; n=18,332 in 2020), intensive care unit (ICU; median 19; IQR: 9-465; n=52,570 in 2019 and median 22; IQR 10-45; n=48,305 in 2020). n=21 outlier above 150% relative change are not shown.*
